# Examining Eating Attitudes and Behaviors in Collegiate Athletes, the Association Between Orthorexia Nervosa and Eating Disorders

**DOI:** 10.3389/fnut.2021.763838

**Published:** 2021-11-11

**Authors:** Nancy A. Uriegas, Zachary K. Winkelmann, Kelly Pritchett, Toni M. Torres-McGehee

**Affiliations:** ^1^Department of Exercise Science, University of South Carolina, Columbia, SC, United States; ^2^Department of Health Sciences, Central Washington University, Ellensburg, WA, United States

**Keywords:** orthorexia, disordered eating, eating attitudes, pathogenic behaviors, eating disorder, athlete

## Abstract

**Purpose:** Orthorexia nervosa (Orthorexia) is an eating attitude and behavior associated with a fixation on healthy eating, while eating disorders (EDs) are clinically diagnosed psychiatric disorders associated with marked disturbances in eating that may cause impairment to psychosocial and physical health. The purpose of this study was to examine risk for Orthorexia and EDs in student-athletes across sex and sport type and determine the association between the two.

**Methods:** Student-athletes (*n* = 1,090; age: 19.6 ± 1.4 years; females = 756; males = 334) completed a survey including demographics, the ORTO-15 test (<40 and <35 threshold values), the Eating Attitudes Test-26 (EAT-26; >20 score), and additional questions about pathogenic behaviors to screen for EDs.

**Results:** Using a <40 threshold value for the ORTO-15, 67.9% were at risk for Orthorexia, a more restrictive threshold value of <35 determined 17.7% prevalence across student-athletes with significant differences across sex [ <40: χ${}_{\left(1,1,090\right)}^{2}$ = 4.914, *p* = 0.027; <35: χ${}_{\left(1,1,090\right)}^{2}$ = 5.923, *p* = 0.015). Overall, ED risk (EAT-26 and/or pathogenic behavior use) resulted in a 20.9% prevalence, with significant differences across sex (χ^2^ = 11.360, *p* < 0.001) and sport-type category (χ^2^ = 10.312, *p* = 0.035). Multiple logistic regressions indicated a significant association between EAT-26 subscales scores and Orthorexia, and between Orthorexia positivity, ORTO-15 scores, and risk for EDs.

**Conclusions:** Risk for Orthorexia and ED is present in collegiate student-athletes. While healthy and balanced eating is important, obsessive healthy eating fixations may increase the risk for EDs in athletes. More education and awareness are warranted to minimize the risk for Orthorexia and EDs in student-athletes.

## Introduction

The emphasis of a “thin ideal” exists in our American society and culture; too often, we see unhealthy messages about ideal weight, body sizes and shapes, attractiveness, and “healthy” eating (1). Unfortunately, often the pressure to maintain a lean physique and ideal body results in increased risk for disordered eating (DE) and feeding and eating disorders (EDs). Both feeding and EDs are serious psychiatric disorders and can result in impaired psychosocial function and physical wellness (2). To date, limited literature exists on the prevalence of feeding disorders in athletes (3), but the prevalence of DE and EDs in athletes has been well-studied across the globe with higher rates observed in athletes as compared with the non-athletic population (4–6).

For young adults including student-athletes, the messages of ideal, lean, and perfect physiques may become pressures and translates to the stereotype that low body weight will increase performance (7), ultimately placing them at risk for EDs. Prevalence of EDs is increasing, especially among female athletes, and while not as prevalent rates in males, it still range from 3.2 to 19.3% across sports (5, 6, 8–12). Over the years, Sundgot-Borgen and Tortstveit have reported gradual increases of ED prevalence in female athletes and non-athletes, from 20 to 28% and 5–21%, respectively (12–15). The increased rates are alarming, and it may be that student-athletes find themselves engaging in pathogenic behaviors (e.g., dieting, restricting, binging, purging, etc.) to reach idealistic weights or the “perfect” body and to optimize performance.

Fortunately, advancements in sports medicine and nutrition have been made, and many collegiate institutions have allied health professionals (e.g., athletic trainers, registered dietitians, team physicians) overseeing the overall health and wellness of athletes. The multidisciplinary health team approach in the career of an athlete is essential. However, at times, the athletes may think allied health professionals do not have their best interest regarding nutrition and performance, so they may seek guidance from unreliable sources, and become obsessed with the quantity and quality of the food they consume. This may become problematic as well; they may want to control their own diet thinking it will enhance their performance and improve recovery (16). Their dietary habits may be restricted, rigid, and obsessive; very much like tendencies associated with orthorexia nervosa (Orthorexia).

While not clinically diagnosed or recognized by the Diagnostic and Statistical Manual of Mental Disorders Fifth Editions (DSM-5) as an ED, Orthorexia has essential characteristics that make it comparable. Across the literature, Orthorexia refers to an obsession with healthy foods and this psychological fixation can often lead to DE behaviors and potentially even clinically diagnosed EDs, if the cases are extreme (17). Its definition was originated from the Greek meaning straight, correct, true appetite, and the modification of the term “anorexia nervosa,” and was originally coined by Dr. Steven Bratman. Dr. Bratman compared Orthorexia to two common EDs, anorexia nervosa and bulimia nervosa, as the three disorders place food as a priority in the scheme of life with the difference that Orthorexia focuses on the quality of food as compared to the quantity (18). While many studies of prevalence of Orthorexia are not reported in the athletic population, those that have been conducted demonstrate high rates in athletes compared with non-athletes (19, 20). Furthermore, utilizing the most common assessment instrument, the ORTO-15, percentages of prevalence among athletes and performing artists range from 56.4 to 88.3%, implementing a threshold value of <40 (19, 21–23). Much debate has occurred regarding the threshold for Orthorexia diagnosis, and literature also suggests a threshold value of <35. Utilizing the <35 threshold value, research on athletes suggests that the prevalence rates of Orthorexia range from 21.5 to 41.4% in all athletes, with varying differences across sex (20, 22, 23).

The concern with Orthorexia is that the behaviors associated may have an association with risk for EDs in athletes. Therefore, the purpose of this study is to assess the risk for Orthorexia utilizing the two proposed threshold values, <40 and <35, and ED in collegiate athletes across sex and sport categories. A secondary aim will look at independent predictors of risk for Orthorexia and the association between risk for Orthorexia and ED risk. We hypothesize that there will be an increased risk for EDs and Orthorexia in athletes, with females expressing higher rates. Utilizing the <40 threshold value for the ORTO-15 will categorize more athletes at risk as compared with the <35 threshold value. Additionally, we hypothesize that pathogenic behavior use and dieting will be indicators of risk for Orthorexia, and risk for Orthorexia will have a positive association with ED risk.

## Materials and Methods

### Study Design

We utilized a cross-sectional study design. The study was part of two larger web-based survey studies. The portion used for this study was descriptive in nature and used a web-based survey developed from previously validated instruments for quantitative analysis. To best assess the risk for Orthorexia and eating attitudes and behaviors, we utilized the ORTO-15 and the Eating Attitudes Test-26 (EAT-26) questionnaires.

### Participants

Collegiate athletes from National Collegiate Athletic Association (NCAA) Division I and II institutions were recruited to participate in the study *via* a web-based survey. A total of *n* = 1,090; age: 19.6 ± 1.4 years; females = 756; males = 334 from across 40 institutions completed the study. To be included in the study, all participants had to be on an active roster during the time of completion of the survey and at least 18 years old. This study was approved by the Institutional Review Board, and prior to completing the survey, all participants consented to the study and had the option to withdraw at any given time.

### Instrumentation

#### Demographic Information

As part of the web-based survey, we collected basic personal and demographic information, including age, sex, race/ethnicity, academic status, sport, self-reported height, and weight (current, lowest, highest, and ideal). Academic status was classified as freshman/1st year college students, sophomores/2nd year college students, juniors/3rd year college students, and seniors/4th or 5th year college students and graduate students. Participants provided their primary sport and these were classified into sport-type categories using Sundgot-Borgen's (13) prior classification of sports: endurance (e.g., cross country, track middle and long distance, swimming), aesthetic (e.g., cheerleading, diving, dance, equestrian), power (e.g., football, track sprints, throws), ball/team (e.g., baseball, softball, basketball, soccer, volleyball, beach volleyball, lacrosse), and technical sports (e.g., golf, tennis, track jumps).

#### ORTO-15

The ORTO-15 diagnostic tool was utilized to assess the risk for Orthorexia in participants (24). The ORTO-15 is a self-administered questionnaire composed of 15 multiple-choice items, and participants answered always, often, sometimes, or never. Answers indicative of orthorexic behaviors were given a score of 1, compared to answers of healthier behaviors having a score of 4. It is the most widely used instrument to evaluate for Orthorexia; however, valid diagnostic criteria remain controversial as this is not a disorder recognized by the DSM-5. The ORTO-15 was adapted from the previously existing Bratman's Orthorexia test (18), and assesses one's attitudes toward selecting, purchasing, preparing, and consuming foods that are considered as healthy or “maniacal obsession for healthy foods” (24). The total score is computed by the summation of all answers with scores ranging from 15 to 60 points; lower scores are indicative of higher levels of attitudes and behaviors associated with Orthorexia and higher scores indicate normal eating behaviors.

Various threshold values have been tested to give a diagnosis of Orthorexia; the two most common are <40 and <35. At a threshold value <40, Donini et al. (24) reported substantial validity of the tool (sensitivity = 100%, specificity = 73.6%, positive predictive value = 17.6, negative predictive value = 100%), with recommendations that a high sensitivity is needed for screening purposes. Whereas, high specificity is required for diagnosis purposes, with a <35 threshold value having 94.3% specificity and 0% sensitivity in the same validation study (24). We assessed prevalence of risk for Orthorexia using both threshold values, the <40 as a risk screening, and the <35 threshold value as a diagnosis, which was entered into further statistical analyses. While originally developed in Italian, the English version was utilized in the present study, as adapted by Donini et al. (24). Further validity and reliability studies have been conducted, suggesting that the ORTO-15 has good repeatability and a reliability deemed satisfactory (Cronbach's alpha = 0.7–0.9) (25). The reliability for the present study was calculated to be α = 0.81.

#### Eating Attitudes Test-26

Participants completed the EAT-26 as a screening tool for EDs characteristics and behaviors (26). The EAT-26 is a self-administered assessment sensitive to different EDs, with a reliability (internal consistency) of α = 0.90 (26). The present study has a Cronbach's alpha of 0.92. While this is not a diagnostic tool, it allows clinicians to identify characteristics and behaviors associated with EDs early. The EAT-26 consists of three subscales: dieting, bulimia, and food preoccupation/oral control and includes five additional questions regarding the use of pathogenic behaviors (e.g., binge eating, vomiting, use of laxatives, diet pills or diuretics, excessive exercise, loss of 9.07 kg (20 lbs) or more in the last 6 months) to lose or control weight. Supplemental questions are scored on a six-point Likert scale (1 = never, 2 = once a month or less, 3 = 2–3 times per month, 4 = once per week, 5 = 2–6 times per week, 6 = once a day or more) and the last question is answered either yes or no. Participants were considered “At Risk” for an ED if they scored >20 points on the EAT-26 and/or if they engaged in at least one pathogenic behavior to lose or control weight, in accordance to guidelines provided by Garner et al. (26).

### Procedures

After approval from the institutional review board, we used a snowball sampling method to recruit participants for the study. We contacted athletes at NCAA Division I and II institutions via their athletic trainers, who received an invitation email containing the link to the survey and were asked to forward the invitation to their athletes. The athletes were able to access the web-based survey via SurveyMonkey (San Mateo, CA) for 1 month, with reminder emails for participation sent every 10 days, for a total of three reminders. The survey included the invitation/consent letter, following consent participants completed the basic demographic survey, the ORTO-15, and the EAT-26, with availability to withdraw from the study at any time point.

### Statistical Analysis

Statistical analyses were conducted using Statistical Package for the Social Sciences (SPSS) (Version 27, SPSS Inc., Armonk, NY) with a significant level set at *p* < 0.05. We carried out two *a priori* power analyses using G^*^Power statistical software (version 3.1.9.2., Heinrich Heine University, Dusseldorf, Germany). The first was used for sex; using an alpha of 0.05 and moderate effect size (0.3), our power calculation indicated a sample of 440 (females = 220, males = 220) with estimated power being 0.95; for sport type, using a large effect size (0.4), our power calculation indicated a sample size of 132 participants (females = 66, males = 66) was needed with estimated power yielding 0.90. Basic descriptive statistics, including means, SD, and frequencies, were used to examine all demographic information (e.g., height, weight, age, calculated body mass index, sex, academic status, etc.), the EAT-26 total score and subscales, and the ORTO-15 total score. A one-way ANOVA was used to compare EAT-26 total score, subscale scores, and ORTO-15 scores with sex and sport-type category. Overall, risk for Orthorexia was considered if participants had a total score <40 or 35 (dependent on threshold value) on the ORTO-15, and ED risk was considered if participants had a score >20 on the EAT-26 and/or they met the criteria for at least one pathogenic behavior (26). A combined and sex-specific crude univariate analysis was conducted to determine the differences between two proportion for risk for EDs given the risk for Orthorexia (score <35) and no risk. Lastly, a multiple logistic regression was conducted to identify independent indicators for risk for Orthorexia, as well as to determine if risk for Orthorexia and EAT-26 subscales were indicators of risk for EDs using the combined total and stratified by sex and controlling for sport-type category.

## Results

A total of 1,105 student-athletes began the study and 1,090 completed the study, yielding a completion rate of 98.6% (females: *n* = 756, age =19.6 ± 1.4 years; males: *n* = 334, age = 19.8 ± 1.4 years) from 40 NCAA Division I and II institutions, meeting the estimated power calculation. Academic status for the participants was as follows: freshman: *n* = 303, 27.8%, sophomore: *n* = 284, 26.1%, juniors: *n* = 263, 24.1% seniors: *n* = 240, 22%. The athletes participated across a total of 26 sports, which were categorized using Sundgot-Borgen's type of sports guidelines: endurance 32.8%, aesthetic 18.8%, power 6.5%, ball/team 34.4%, and technical sports 7.5%. Self-reported physical measures are presented in Table 1.

**Table 1:** Descriptive statistics for participant demographics and self-reported anthropometrics.

	**All (*n* = 1,090)**	**Endurance (*n* = 357)**	**Aesthetic (*n* = 205)**	**Power (*n* = 71)**	**Ball/team (*n* = 375)**	**Technical (*n* = 51)**
**Females**	***n* = 755**	***n* = 250**	***n* = 181**	***n* = 23**	***n* = 251**	***n* = 51**
Age (years)	19.6 ± 1.4	19.9 ± 1.4	19.6 ± 1.3	20.0 ± 1.1	19.3 ± 1.3	19.5 ± 1.2
Weight (kg)
Current	64.5 ± 10.6	64.1 ± 9.8	59.3 ± 8.1	70.6 ± 19.4	68.4 ± 10.8	62.3 ± 7.0
Highest	67.2 ± 11.2	66.8 ± 10.0	61.9 ± 87	73.7 ± 20.5	71.1 ± 11.6	65.6 ± 7.5
Lowest	60.0 ± 9.7	59.4 ± 8.9	55.3 ± 7.4	65.3 ± 17.0	63.7 ± 10.0	58.8 ± 6.4
Ideal	61.9 ± 9.3	61.6 ± 8.3	56.7 ± 6.6	69.0 ± 17.2	65.6 ± 9.6	60.1 ± 5.6
Current-ideal	2.6 ± 3.8	2.5 ± 3.1	2.6 ± 3.2	1.7 ± 4.0	2.8 ± 4.9	2.2 ± 2.3
Height (cm)	168.8 ± 7.8	19.7 ± 7.0	164.1 ± 6.5	171.7 ± 5.4	171.3 ± 8.6	167.7 ± 5.4
BMI (kg/m^2^)	22.6 ± 2.9	22.2 ± 2.6	22.0 ± 2.4	23.9 ± 5.8	23.3 ± 3.0	22.1 ± 1.2
**Males**	***n*** **=** **334**	***n*** **=** **107**	***n*** **=** **24**	***n*** **=** **48**	***n*** **=** **124**	***n*** **=** **31**
Age (years)	19.8 ± 1.4	19.8 ± 1.4	20.6 ± 1.8	19.7 ± 1.3	19.5 ± 1.3	30.0 ± 1.3
Weight (kg)
Current	83.1 ± 13.3	77.5 ± 8.4	84.0 ± 20.7	97.4 ± 16.9	82.9 ± 10.4	79.5 ± 7.2
Highest	85.9 ± 14.8	80.6 ± 9.7	84.6 ± 22.5	100.8 ± 19.1	85.8 ± 11.8	82.1 ± 9.5
Lowest	77.8 ± 12.6	72.8 ± 8.1	77.2 ± 20.6	89.9 ± 15.3	78.2 ± 10.5	75.5 ± 7.0
Ideal	85.0 ± 13.1	79.3 ± 8.8	83.2 ± 17.6	99.3 ± 17.7	85.7 ± 9.8	80.6 ± 6.2
Current-Ideal	−1.9 ± 4.2	−1.8 ± 3.8	0.8 ± 5.5	−1.9 ± 5.4	−2.8 ± 3.8	−1.1 ± 2.7
Height (cm)	183.5 ± 7.2	183.5 ± 7.3	181.2 ± 7.0	185.3 ± 6.4	193.1 ± 7.7	184.1 ± 4.7
BMI (kg/m^2^)	24.6 ± 3.2	23.0 ± 1.7	25.4 ± 5.3	28.3 ± 4.0	24.7 ± 2.2	23.5 ± 1.8

*Data is expressed as mean , SD*.

### Risk for Orthorexia Prevalence

The risk for Orthorexia was assessed using the two established threshold values for the ORTO-15, <40 and <35, significant differences were found across sex for both the threshold values <40: [χ${}_{\left(1,1,090\right)}^{2}$ = 4.914, *p* = 0.027], <35: [χ${}_{\left(1,1,090\right)}^{2}$ = 5.923, *p* = 0.015]. Comparisons across sport types and sex resulted in no significant differences (*p* = 0.462), assessing through individual sport type and sex significant differences were found for technical [ <40: χ${}_{\left(1,82\right)}^{2}$ = 4.495, *p* = 0.034] and ball/team [ <35: χ${}_{\left(1,375\right)}^{2}$ = 5.287, *p* = 0.021]. Mean scores and SDs for the ORTO-15 and frequencies for “at-risk” are presented on Table 2.

**Table 2:** Risk for Orthorexia across collegiate athletes using ORTO-15.

	**Raw score Mean ± SD**	**Score <40 % (n)**	**Score <35 % (n)**
All participants (*n* = 1,090)	37.7 ± 3.7^*^	67.9 (741)^*^	17.7 (193)^*^
Female (*n* = 756)	37.5 ± 3.8	69.9 (529)	19.6 (148)
Male (*n* = 334)	38.2 ± 3.4	63.3 (212)	13.4 (45)
**Sport type**
Endurance (*n* = 357)	37.6 ± 3.7	70.3 (251)	17.9 (64)
Female	37.3 ± 3.6	72.4 (181)	18.8 (47)
Male	38.2 ± 3.8	65.4 (70)	15.9 (17)
Aesthetic (*n* = 205)	37.8 ± 3.8	65.4 (134)	16.1 (33)
Female	37.7 ± 3.9	65.7 (119)	17.7 (31)
Male	38.5 ± 3.3	62.5 (15)	8.3 (2)
Power (*n* = 71)	38.3 ± 3.4	60.6 (43)	12.7 (9)
Female	38.1 ± 3.4	69.6 (16)	13.0 (3)
Male	38.4 ± 3.5	56.3 (27)	12.5 (6)
Ball/team (*n* = 375)	37.7 ± 3.7	68.8 (258)	20.5 (77)^*^
Female	37.5 ± 4.0	69.7 (175)	23.9 (60)
Male	38.1 ± 2.9	66.9 (83)	13.7 (17)
Technical (*n* = 82)	37.5 ± 3.6	65.9 (54)^*^	12.2 (10)
Female	37.0 ± 3.3	74.5 (38)	13.7 (7)
Male	38.5 ± 4.0	51.6 (16)	9.7 (3)

*Data is presented in means, SDs, frequencies, and percentages*.

* *p-value < 0.05*.

### Prevalence of ED Risk and Pathogenic Behaviors

Table 3 depicts risk for EDs and mean scores for EAT-26 and all subscales (i.e., diet, bulimia, oral control) across sex and sport type. Overall assessing the combined risk for EDs (EAT-26 and/or use of pathogenic behaviors), 20.9% (*n* = 228) of all athletes were at risk with significant differences across sex [χ${}_{\left(1,1,090\right)}^{2}$ = 11.360, *p* < 0.001] and sport type [χ${}_{\left(4,1,090\right)}^{2}$ = 10.312, *p* = 0.035]. Comparison across sport type and within sex revealed significant differences in mean EAT-26 scores for endurance (*p* = 0.018), aesthetic (*p* = 0.009), and ball/team (*p* < 0.001) sports with females scoring higher than males. Participants that were found at risk were categorized by ED risk type (EAT-26 only, pathogenic behaviors only, or both EAT-26 and pathogenic behaviors) with significant differences across sex [χ${}_{\left(3,1,090\right)}^{2}$ = 17.944, *p* < 0.001] and sport type [χ$[\chi_{(\text{12},\text{1},\text{090}}^{2}$ = 29.579, *p* = 0.003]. Looking at the sport-type alone, there were significant differences for endurance and sex [χ${}_{\left(3,357\right)}^{2}$ = 14.441, *p* = 0.002], and no significant differences for sex and aesthetic, power, ball/team, or technical sports.

**Table 3:** Risk for EDs across collegiate athletes using EAT-26 and pathogenic behaviors.

	**Raw scores**				**ED risk and type**			
	**mean** **±** **SD**				**% (** ***n*** **)**			
	**EAT-26**	**Diet subscale**	**Bulimia subscale**	**Oral control**	**Overall risk**	**EAT-26 only**	**Behavior only**	**Both**
All participants	6.1 ± 8.0	3.9 ± 5.5	0.7 ± 2.1^*^	1.6 ± 2.1	20.9 (228)^*^	1.4 (15)	15.2 (166)	4.3 (47)
Female (*n* = 756)	6.9 ± 8.2	4.6 ± 5.9	0.8 ± 2.0	1.6 ± 2.0	23.7 (179)	1.6 (12)	16.3 (123)	5.8 (44)
Male (*n* = 334)	4.4 ± 7.1	2.4 ± 4.1	0.4 ± 1.8	1.6 ± 2.3	14.6 (49)	0.9 (3)	12.8 (43)	0.9 (3)
**Sport type**								
Endurance (*n* = 357)	6.5 ± 9.2^*^	4.0 ± 6.0^*^	1.0 ± 2.4	1.6 ± 2.4	26.3 (94)^*^	0.3 (1)	19.3 (69)	6.7 (24)
Female	7.3 ± 9.3	4.7 ± 6.4	1.1 ± 2.5	1.5 ± 2.2	30.8 (77)	0 (0)	21.6 (54)	9.2 (23)
Male	4.8 ± 8.7	2.4 ± 4.4	0.6 ± 2.3	1.8 ± 2.8	15.7 (17)	0.9 (1)	14.0 (15)	0.9 (1)
Aesthetic (*n* = 205)	7.0 ± 8.3^*^	4.7 ± 5.7^*^	0.7 ± 2.3	1.5 ± 2.0	17.1 (35)	2.0 (4)	11.7 (24)	3.4 (7)
Female	7.5 ± 8.7	5.2 ± 5.9	0.8 ± 2.5	1.6 ± 2.1	17.7 (32)	2.2 (4)	11.6 (21)	3.9 (7)
Male	2.8 ± 2.2	1.5 ± 1.9	0.2 ± 0.4	1.1 ± 1.1	12.5 (3)	0 (0)	12.5 (3)	0 (0)
Power (*n* = 71)	5.9 ± 9.5	3.6 ± 5.8	0.5 ± 2.5	1.8 ± 2.7	15.5 (11)	1.4 (1)	14.1 (10)	0 (0)
Female	5.7 ± 4.6	3.3 ± 3.1	0.5 ± 2.1	1.9 ± 2.2	17.4 (4)	0 (0)	17.4 (4)	0 (0)
Male	6.0 ± 11.1	3.7 ± 6.7	0.5 ± 2.7	1.8 ± 2.9	14.6 (7)	2.1 (1)	12.5 (6)	0 (0)
Ball/team (*n* = 375)	5.5 ± 6.4^*^	3.5 ± 4.9^*^	0.5 ± 1.4^*^	1.6 ± 1.8	18.9 (71)	1.3 (5)	13.6 (51)	4.0 (15)
Female	6.3 ± 7.1	4.1 ± 5.5	0.7 ± 1.6	1.6 ± 1.8	21.5 (54)	2.0 (5)	14.3 (36)	5.2 (13)
Male	3.9 ± 4.0	2.2 ± 2.9	0.1 ± 0.5	1.6 ± 1.9	13.7 (17)	0 (0)	12.1 (15)	1.6 (2)
Technical (*n* = 82)	5.5 ± 6.4	3.5 ± 4.5^*^	0.7 ± 1.8	1.3 ± 1.8	20.7 (17)	4.9 (4)	14.6 (12)	1.2 (1)
Female	6.6 ± 7.2	4.5 ± 5.2	0.7 ± 1.7	1.4 ± 2.1	23.5 (12)	5.9 (3)	15.7 (8)	2.0 (1)
Male	3.8 ± 4.3	1.9 ± 2.1	0.7 ± 2.0	1.3 ± 1.3	16.1 (5)	3.2 (1)	12.9 (4)	0 (0)

*Data are presented in means, SDs, frequencies, and percentages*.

* *p-value < 0.05*.

A total of 19.6% (*n* = 214) of the participants reported the use of pathogenic behaviors (e.g., binge eating, vomiting, use of diet pills or laxatives, excessive exercise, etc.) to control weight, with females (22.2%, *n* = 168/756) engaging in significantly more pathogenic behaviors than males [13.8%, *n* = 46/334 χ${}_{\left(1,1,090\right)}^{2}$ = 10.483, *p* = 0.001]. When assessing the behaviors individually, significant differences were found between sex and binge eating [χ${}_{\left(1,1,090\right)}^{2}$ = 7.375, *p* = 0.007], use of diet pills/laxatives [χ${}_{\left(1,1,090\right)}^{2}$ = 6.829, *p* = 0.009], and previous ED (χ^2^ = 5.747, *p* = 0.017). No differences were found for vomiting (*p* = 0.114), excessive exercise (*p* = 0.297), or loss of 20l bs (9.1 kg) or more (*p* = 0.810), and sex. Across types of sport differences were reported for binge eating [χ${}_{\left(1,1,090\right)}^{2}$ = 10.935, *p* = 0.027] and excessive exercise [χ${}_{\left(1,1,090\right)}^{2}$ = 27.630, *p* < 0.001], with a higher percentage of endurance athletes reporting these behaviors.

### Associations Between Risk for Orthorexia and Risk for EDs

A crude univariate analysis of the differences between proportions indicated that across the entire athlete sample, there is a 20.6% greater risk of being at risk for EDs in athletes who are at risk for Orthorexia (using a threshold value <35) as compared with those who are not at risk for Orthorexia (*p* < 0.001). When stratified by sex, females and males at risk for Orthorexia had a 25.1% (*p* < 0.001) and 16.4% (*p* = 0.004) greater risk of being at risk for EDs compared with those who were not at risk for Orthorexia, respectively.

Multiple logistic regression analyses for independent predictors of risk for Orthorexia are presented in Table 4, controlled by sport type. Significant positive associations were found for the EAT-26 subscales (diet, bulimia, oral control) and use of pathogenic behaviors across the total sample and stratified by sex. Previous ED was positively associated with Orthorexia for the total sample and females only. No significant associations were found for current and ideal weight or BMI.

**Table 4:** Logistic regressions analysis for the presence of ON risk, stratified by sex controlled for sport type.

	**Exp (B)**	**95% CI**	***p-*value**
**Total (*****n*** **=** **1,090)**			
Diet subscale	1.16	1.13–1.19	<0.001
Bulimia subscale	1.29	1.20–1.39	<0.001
Oral control subscale	1.16	1.08–1.24	<0.001
Pathogenic behavior risk	2.82	1.99–4.00	<0.001
Previous ED	3.56	1.54–8.21	0.003
**Females (*****n*** **=** **756)**			
Diet subscale	1.17	1.13–1.21	<0.001
Bulimia subscale	1.32	1.22–1.44	<0.001
Oral control subscale	1.18	1.09–1.28	<0.001
Pathogenic behavior risk	2.91	1.95–4.33	<0.001
Previous ED	3.89	1.63–9.27	0.002
**Males (*****n*** **=** **334)**			
Diet subscale	1.11	1.04–1.18	0.002
Bulimia subscale	1.15	1.01–1.31	0.041
Oral control subscale	1.12	1.00–1.25	0.045
Pathogenic behavior risk	2.33	1.08–5.02	0.031

Table 5 includes the independent predictors for overall ED risk, controlled for sport type. Overall, ORTO-15 positivity (“at-risk”) and ORTO-15 scores had a significant association (*p* < 0.001) with ED risk for the total sample of athletes and across sex. Additionally, significant associations were found for all subscales of the EAT-26 across all participants and across sex categories.

**Table 5:** Logistic regressions analysis for the presence of ED risk, stratified by sex controlled for sport type.

	**Exp (B)**	**95% CI**	***p-*value**
**Total (*****n*** **=** **1,090)**			
ORTO-15 positivity	3.43	2.44–4.82	<0.001
ORTO-15 score	0.85	0.82–0.89	<0.001
Diet subscale	1.30	1.25–1.36	<0.001
Bulimia subscale	2.31	1.98–2.69	<0.001
Oral control subscale	1.16	1.09–1.24	<0.001
**Females (*****n*** **=** **756)**			
ORTO-15 positivity	3.52	2.37–5.20	<0.001
ORTO-15 score	0.84	0.85–0.89	<0.001
Diet subscale	1.31	1.25–1.37	<0.001
Bulimia subscale	2.53	2.10–3.06	<0.001
Oral control subscale	1.14	1.06–1.24	<0.001
**Males (*****n*** **=** **334)**			
ORTO-15 positivity	2.85	1.37–5.97	0.005
ORTO-15 score	0.90	0.83–0.98	0.017
Diet subscale	1.29	1.15–1.46	<0.001
Bulimia subscale	1.65	1.22–2.23	0.001
Oral control subscale	1.21	1.07–1.37	0.002

*ED risk, Score >20 points and/or engagement in pathogenic behaviors*.

## Discussions

The health implications associated with DE and EDs typically result in negative performance outcomes, ultimately having the opposite effect than what the athlete expected (7). Limited studies have been conducted on Orthorexia prevalence in athletes, most being in recreational athletes, with no studies conducted in the United States. To our knowledge, only one study, by Clifford and Blyth (19), has been conducted specifically on prevalence of Orthorexia in collegiate athletes in the United Kingdom, but no specifics were provided if they participated in recreational/intramural or collegiate “varsity” sports. Therefore, this would make this the first study to examine risk for Orthorexia in collegiate athletes in the United States. The purpose of this study was to assess the prevalence of risk for Orthorexia and risk for EDs in collegiate athletes and determine the association between both. As expected, the findings suggest that the prevalence of risk for Orthorexia and EDs exists in athletes with higher rates among females than males. These findings are consistent with previous studies conducted on Orthorexia in athletes (20, 23, 27), as well as those conducted on risk for EDs and athletes, without formal interviews (12, 28). Moreover, athletes are engaging in pathogenic behaviors to control their weight, which our findings indicated as a significant association with risk for Orthorexia.

### Prevalence of Risk for Orthorexia

When assessing risk for Orthorexia, we utilized two threshold values, <40 and <35, for the ORTO-15, given the controversies associated with the higher threshold. Donini et al. (24) suggested that the <40 threshold value with high sensitivity should be utilized as a screening method, while the <35 threshold value with high specificity should be used for diagnostic purposes. However, it should be noted that the DSM-5 does not recognize Orthorexia as a mental illness. Utilizing the <40 threshold value for screening, we identified a high percentage, 67.9%, of athletes at risk for Orthorexia, female at 69.9% and males at 63.3%. Previous studies assessing Orthorexia that used this same threshold have reported rates ranging from 56.4 to 88.3%, consistent with our findings (19, 21–23, 27). Furthermore, these results are closely related to those found in recreational athletes aged 18–40 years, exercising <150 min (71.1%) and >150 min (72.8%) per week (22). Student-athletes face increased exercise energy expenditure, and 150 min may represent the physical activity conducted in one practice session, for a total of at least 12.5 h a week, this resulting in increased energy demands to maintain energy balance. Lastly, in comparison to the study conducted in university athletes (19), the risk in our sample appeared to be lower by ~8% (76 vs. 68%), this can be due to the difference in sample size, as our study had 1,090 participants and could be more generalizable to the population.

However, when using a more restrictive threshold value of <35, the prevalence rates in our sample decreased to 17.7%. These rates are lower in comparison to Segura-Garcia et al. (20) and Surala et al. (23), who assessed the prevalence of Orthorexia using the ORTO-15 <35 threshold value, with results indicating positivity in 28% of athletes and 41.4% of Olympic sport competitive athletes, respectively. Our findings and those in studies conducted in athletes ultimately suggest that Orthorexia attitudes and behaviors are present in the athletic population. Agreement has not been determined regarding sex differences in Orthorexia, with varying studies reporting higher prevalence in males, while others report higher prevalence in females. We identified that females have a higher rate of Orthorexia (19.6%) as compared to males (13.4%), aligning with findings by Herranz Valera et al. (27) and Segura-Garcia et al. (20) in athletes but also with studies conducted in non-athlete populations (29, 30).

With regards to sport-type categories, no studies have assessed Orthorexia to determine differences across sport type. However, Clifford and Blyth (19) did stratify their sample size by weight-dependent sports, finding that 41% of weight-dependent athletes were at risk compared with 59% of athletes in non-weight-dependent sports. Further assessment determined that females in weight-dependent sports have higher risk (74%) compared to males (16%). While we did not stratify by weight dependence, prevalence across sport-type categories existed. Higher rates were observed in females participating in endurance and ball/team sports. Ball/team sports included volleyball and beach volleyball, two sports where debate exists if they should be ball/team or aesthetic sports.

### Prevalence of Risk for EDs and Pathogenic Behaviors

We determined the overall risk for EDs (EAT-26 and/or pathogenic behavior use) across athletes to be 20.9%. These findings are consistent with the studies conducted on both female and male athletes together, where screening for ED risk resulted in 14.5–25% prevalence (10, 12). There are few studies conducted specifically on male athletes, however, some do include them in their sample of interests. Those studies report rates of 3.2-19.2% across male athletes, which are consistent with the findings from our studies where male athletes had a 14.6% overall risk for ED (5, 8–10, 12). Sex differences exist in EDs, with higher rates (11–32.8%) typically reported for females, with increases over the years (5, 6, 8, 12–15, 31).

Taking a further look into EDs in females, Sundgot-Borgen (13) categorized athletes based on the demands of their sports (endurance, aesthetic, power, weight-dependent, ball/team, and technical), and reported prevalence accordingly. To date, most studies will categorize athletes based on the leanness or weight-dependence of their sport (lean/weight-dependent vs. non-lean/non-weight-dependent), especially throughout female sports. This study looked at sex and sport-type differences, comparisons can be made Sundgot-Borgen's 1993 study (13) if we look strictly at females. However it is important to note the difference in assessment tools used in both studies, we utilized the EAT-26 while they used the EDI-2, pathogenic behaviors and formal interviews. When looking at the findings based on the assessment tools only (EDI-2 vs. EAT-26) from their studies compared with ours, the differences are as follows: endurance 20 vs. 9.2%, aesthetic 34 vs. 6.1%, ball/team 11 vs. 7.2%, technical 13 vs. 7.2%, no differences in power sport type and we did not conduct studies on the weight-dependent category (13). Differences exist but these can be due to the difference in the assessment tool, given that the EDI includes both traditional and comorbid psychological factors (32).

### Associations Between Orthorexia and EDs

We determined that there is a significant greater risk of 20.6% of risk for EDs in athletes who are at risk for Orthorexia as compared with those who are not at risk for Orthorexia. Furthermore, regression demonstrated the association between pathogenic behaviors, previous ED, and EAT-26 subscales to Orthorexia and Orthorexia positivity and ORTO-15 scores to ED. Studies have assessed the association of factors like BMI, minutes of sport, self-reported current or past ED, and sex to Orthorexia with significant findings (20, 22, 23, 33). Segura-Garcia et al. (20) included factors like previous dieting and the need to purge in their analyses; these two factors can be considered as pathogenic behaviors, which had a significant association with Orthorexia in the present study. Moreover, there were associations between self-reported current and past ED and Orthorexia in university students in Poland, Spain, and Italy (33). While this sample was not athletes, their age range is similar, and they may also be facing some of the stressors associated with attending college in a different area, like student-athletes.

With regards to the association between Orthorexia and ED, studies have looked at the impart of ED positivity and DE behaviors on Orthorexia but not Orthorexia as a factor impacting EDs. In 2019, Dunn et al. (34) reported that individuals who self-identified as having Orthorexia, scored in the clinical range of the EAT-26. No assessment tool was used in their study to determine the presence of Orthorexia as was done in this study, with similar findings of positivity in the EAT-26 and/or presence of pathogenic behaviors.

### Clinical Implications

Collegiate athletes face physical and mental stressors associated with athletic performance and being a student, potentially in a new place, without their typical support system. These stressors may drive their decision-making of health and wellness. Unfortunately, student-athletes will utilize the information most readily accessible, typically found on the internet or through unreliable sources, which ultimately may predispose them to unhealthy behaviors. A multidisciplinary health team approach is imperative in organized sports to provide athletes with adequate and reliable information. This team can be composed of athletic trainers, registered dietitians, mental health specialists, and team physicians. Athletic trainers focus on injury and illness prevention, diagnosis, and management, but may also engage with athletes regarding nutrition and performance. On the other hand, the role of dietitians is more extensive and includes assessment of dietary needs, prescription, and education in food selection, purchasing, and preparation (16). The commonalities shared by these allied healthcare professionals are the health, wellness, and safety of the athlete by providing education, prevention strategies, and adequate interventions for those presenting with DE attitudes and behaviors, including Orthorexia.

The present study identified a higher risk for both Orthorexia and EDs in females, which has been widely acknowledged by the literature. We further identify females in endurance sports have a higher risk for EDs than other sport-type categories and their male counterparts. Similarly, females in ball/team sports have a higher risk for Orthorexia than males in the same sport type. This is a key information for clinicians who may consider screening athletes using validated tools such as the EAT-26 or ORTO-15, to assess eating attitudes and behaviors to determine risk for EDs. They may implement screening techniques on those athletes who have higher risk and can implement education and early interventions to minimize the decline in their overall health and wellness.

### Limitations

Limitations exist within the present study. We must note that both the EAT-26 and the ORTO-15 are previously validated instruments, however, they are self-reported measures, and participants may not provide true and honest responses. Additionally, both instruments are used as an assessment of eating attitudes and behaviors, they are not utilized to diagnose EDs or Orthorexia. The gold standard for clinically diagnosed EDs is interviews conducted by a mental health provider, and Orthorexia is currently not a diagnosable mental illness. Future research should explore the use of interviews for those at risk for EDs and Orthorexia for definitive diagnoses. Furthermore, the use of dietary records, dietary recall logs, or food frequency logs can be implemented to include more detailed information on the foods being consumed.

## Conclusions

Reflecting on the use of different thresholds for the ORTO-15 tool and their original meaning, a threshold value <40 can be used as a screening measure. Given that information, the risk for Orthorexia is highly prevalent for female and male collegiate athletes in the United States. However, when implementing stricter guidelines (threshold value <35), we observe the risk diminishes but it is still present. Moreover, a high percentage of athletes reported to have been engaging in pathogenic behaviors to control their weight, with females displaying higher risk. The concern exists that pathogenic behaviors can be associated with Orthorexia. Our findings further reveal that Orthorexia can be associated with the risk of developing an ED, which is also prevalent among the athletic population. It is crucial to provide athletes with education programs as methods of prevention. These should focus on reduction of stigma, promotion of healthy relationships with food and one's body, adequate nutritional strategies, and the potential of health and performance consequences associated with EDs and DE (35). Additionally, initial comprehensive and continuing education should be provided to coaches, support staff, and allied healthcare professionals working with athletes (35). While encouraging healthy eating behaviors and high-performance fueling strategies is important, one must remember to not be fixated on healthy diets but instead have flexible eating patterns and nutritional strategies.

## Data Availability Statement

The raw data supporting the conclusions of this article will be made available by the authors, without undue reservation.

## Ethics Statement

The studies involving human participants were reviewed and approved by University of South Carolina Institutional Review Board. The patients/participants provided their written informed consent to participate in this study.

## Author Contributions

TT-M was involved in the design of the study and collected the data. NU analyzed the data. NU, ZW, KP, and TT-M contributed to drafts and approved the final manuscript. All authors contributed to the article and approved the submitted version.

## Conflict of Interest

The authors declare that the research was conducted in the absence of any commercial or financial relationships that could be construed as a potential conflict of interest.

## Publisher's Note

All claims expressed in this article are solely those of the authors and do not necessarily represent those of their affiliated organizations, or those of the publisher, the editors and the reviewers. Any product that may be evaluated in this article, or claim that may be made by its manufacturer, is not guaranteed or endorsed by the publisher.
